# Supplementary material for: Palliative Care Landscape in the COVID-19 Era: Bibliometric Analysis of Global Research
Source: Healthcare (Basel). 2022 Jul 20;10(7):1344. doi: 10.3390/healthcare10071344 (PMC9318933; doi:10.3390/healthcare10071344)
Supplement: Supplementary file 1 [file healthcare-10-01344-s001.zip › healthcare-1811620-supplementary.pdf]

Table S1: Scopus search strategies for articles.

**Number**	**Search Term**	**Hits**
Search in Scopus; Date of search: April 25th, 2022		
#1	TITLE ("palliative care" OR "palliative medicine" OR "hospice care" OR "terminal care" OR "end-of-life care" OR "end of life care" OR "palliat*" OR "life-limiting" OR "life-threatening" OR "incurable disease" OR "supportive care")	57,962
#2	ABS ("palliative care" OR "palliative medicine" OR "hospice care" OR "terminal care" OR "end-of-life care" OR "end of life care" OR "palliat*" OR "life-limiting" OR "life-threatening" OR "incurable disease" OR "supportive care")	231,729
#3	#1 AND #2	34,521
#4	TITLE ("novel coronavirus 2019" OR "2019-nCov" OR "2019 Novel Coronavirus" OR "coronavirus" OR "middle east respiratory syndrome" OR "coronavirus disease 2019" OR "coronavirus 2019" OR "COVID 2019" OR "COVID 19" OR "nCOV" OR "SARS" OR "MERS" OR "SARS-CoV-2" OR "COVID-19" OR "COVID*")	316,095
#5	ABS ("novel coronavirus 2019" OR "2019-nCov" OR "2019 Novel Coronavirus" OR "coronavirus" OR "middle east respiratory syndrome" OR "coronavirus disease 2019" OR "coronavirus 2019" OR "COVID 2019" OR "COVID 19" OR "nCOV" OR "SARS" OR "MERS" OR "SARS-CoV-2" OR "COVID-19" OR "COVID*")	377,276
#6	#4 AND #5	234,017
#7	#3 AND #6	720
#8	#7 LIMIT-TO (TYPE, “ARTICLE”	583

Explanation of abbreviations: # = Search; ABS=Abstract
